# Clinical characteristics of patients with *KRAS* mutation detected by liquid biopsy

**DOI:** 10.1111/1759-7714.15123

**Published:** 2023-09-26

**Authors:** Yoshiaki Amino, Siew‐Kee Low, Hironori Ninomiya, Ayu Kiritani, Keiki Miyadera, Sho Kakuto, Takahiro Akita, Ryosuke Tsugitomi, Ryo Ariyasu, Ken Uchibori, Satoru Kitazono, Noriko Yanagitani, Makoto Nishio

**Affiliations:** ^1^ Department of Thoracic Medical Oncology Cancer Institute Hospital of Japanese Foundation for Cancer Research Tokyo Japan; ^2^ Cancer Precision Medicine Center, Japanese Foundation for Cancer Research Tokyo Japan; ^3^ Division of Pathology Cancer Institute, Japanese Foundation for Cancer Research Tokyo Japan; ^4^ Department of Pathology The Cancer Institute Hospital, Japanese Foundation for Cancer Research Tokyo Japan; ^5^ Department of Respiratory medicine Jikei University School of Medicine Tokyo Japan; ^6^ Department of Respiratory medicine Hachinohe City Hospital, Hachinohe Aomori Japan

**Keywords:** *KRAS*, liquid biopsy, next‐generation sequencing, non‐small cell lung cancer

## Abstract

**Background:**

*KRAS* mutation positive lung cancer is known to be clinically characterized by older age, males, and smokers. It is reported to be more common in mucinous adenocarcinoma, but all reports are based on analysis of tissue samples. Recently, blood samples have become available for analysis, suggesting a low detection rate of circulating tumor DNA in histological types, especially mucinous adenocarcinoma. In this study, we investigated the clinical characteristics of *KRAS* mutation‐positive cases in the analysis of blood specimens, as these remain unclear.

**Methods:**

The clinical background of patients with *KRAS* mutation among those who underwent next‐generation sequencing (NGS) analysis using blood samples was evaluated.

**Results:**

NGS analysis was performed on 214 blood samples. *KRAS* mutations were detected in blood samples in 33 cases (15.4%), of which 31 cases (14.5%) had a histological pathology diagnosis. Mucinous adenocarcinoma accounted for 28.6% of cases with positive blood and tissue specimens, 10.0% of cases with positive blood specimens only, and 57.1% of cases with positive tissue specimens only. Mucinous adenocarcinoma tended to be less common in cases with positive blood specimens. In *KRAS*‐positive patients with lung metastasis only, only one nonmucinous adenocarcinoma had a positive blood sample, and the others all had mucinous adenocarcinomas with positive tissue samples only.

**Conclusion:**

The results showed that the detection rate of *KRAS*‐positive lung cancers detected by blood and tissue samples differs, and that the detection rate of blood samples may be poor, especially in the case of mucinous adenocarcinoma with lung metastases only.

## Graphical abstract

The detection rate of *KRAS*‐positive lung cancers analyzed by blood and tissue samples differs, and the detection rate of blood samples may be poor, especially in the case of mucinous adenocarcinoma with lung metastases only.

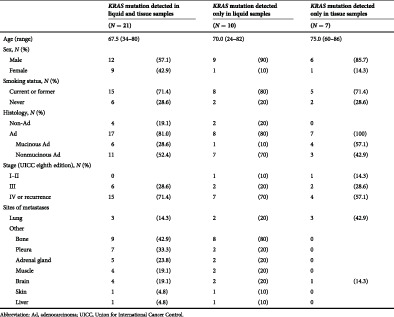

## INTRODUCTION

Lung cancer is the leading cause of death, but it is also the carcinoma with the most recent decline in mortality.^1^ It has been suggested that one of the reasons for the decline in mortality is the approval and introduction of molecularly targeted drugs into practice.^2^ The *KRAS* gene was discovered as an oncogene by Stehelin et al. in 1976,^3^ and although various drugs have been developed since then, many have not shown clinical efficacy and *KRAS* mutation has remained an undruggable target.^4^ *KRAS* mutation frequency was reported to be 22% in an analysis of 500 lung adenocarcinoma cases.^5^ Recently approved molecular targeted agents, sotorasib and adagrasib, *KRAS* G12C inhibitors, are of interest.^6^, ^7^ With the advent of treatment methods, it has become clinically important to identify *KRAS*‐positive lung cancers, especially *KRAS* G12C‐positive lung cancers. *KRAS*‐positive lung cancers have been identified with *KRAS* mutations by tissue‐based analysis. Some clinical features have been reported. Unlike *EGFR* mutation and *ALK* fusion, it is more common in older people, men and smokers.^8^ Invasive mucinous adenocarcinoma is reported to be the most common histological type.^9^ The frequency of *KRAS* mutations in non‐small cell lung cancer (NSCLC) cases has been reported to be 20%–30%, while in invasive mucinous adenocarcinoma cases the frequency is 50%–70%.^10^

Currently, the gold standard for detecting genetic mutation is analysis of tumor tissue samples.^11^ Liquid biopsy has recently received attention as an alternative to tissue biopsy. Liquid biopsy is minimally invasive and has the advantage of a short turnaround time of approximately 7 days compared to 2–3 weeks for tissue biopsy.^12^ However, liquid and tissue biopsy results are not always consistent. The specificity of the plasma circulating tumor DNA (ctDNA) assay is relatively high. On the other hand, the sensitivity is relatively low and false negative rates of up to 30% have been reported.^11^

Causes of discrepancy between next‐generation sequencing (NGS) analysis in liquid and tissue samples are not clear. ctDNA is reported to be frequently low or less sensitive in CNS and mucinous tumors.^13^, ^14^ Nonmucinous adenocarcinoma is more likely to be detected by liquid NGS than mucinous adenocarcinoma.^14^ Knowing the characteristics of *KRAS*‐positive cancers, especially lung cancer that is *KRAS*‐positive with NGS of liquid biopsies (liquid *KRAS*‐positive) lung cancer, might help effectively detect *KRAS*‐positive lung cancers in clinical practice. We analyzed the difference in the *KRAS* mutation detection rate with liquid NGS between nonmucinous and mucinous histological types, including adenocarcinoma. In the present study, we retrospectively examined the clinical characteristics of patients with *KRAS*‐positive lung cancer who underwent plasma NGS analysis using the ION Torrent Genexus System (Genexus)/Oncomine Precision Assay (OPA) in a previous study^15^ conducted at our hospital.

## METHODS

At this hospital between August 2020 and March 2022, NGS analysis was performed on blood samples of 214 NSCLC cases using the ION Torrent Genexus System (Genexus)/OPA. We reviewed the medical background of the patients including age, gender, histology, smoking history, stage, metastatic disease, *KRAS* mutations, and coexpression mutations. Adenocarcinomas were classified into mucinous and nonmucinous histological types. Mucinous adenocarcinomas were defined as composed of columnar and goblet cells with prominent cytoplasmic mucin.

The study was approved by the Ethics Committee of Cancer Institute Hospital of Japanese Foundation for Cancer Research (IRB no.: 2022‐GB‐091). Informed consent was obtained from all individual participants included in the present study. This study was conducted according to the Declaration of Helsinki, seventh version.

NGS analysis was performed using Genexus/OPA, and plasma samples were collected before initial treatment. Samples were collected from 14 mL of blood using EDTA‐2Na collection tubes, and cell free DNA and RNA in the samples were extracted for target sequencing using Genexus.^15^ The Oncomine Dx Target Test Multi‐CDx System (Ion Torrent PGM Dx Sequencer; Thermo Fisher Scientific) (Oncomine DxTT) was approved in Japan in February 2019 for formalin‐fixed paraffin‐embedded and fresh‐frozen specimens for NSCLC, and is an NGS panel that performs analysis using DNA and RNA isolated from fresh‐frozen specimens.^16^ Patients with *KRAS* mutation identified through NGS analysis of plasma samples with Genexus/OPA were designated as liquid *KRAS*‐positive. Patients with *KRAS* mutation identified through NGS analysis of tissue samples with Oncomine DxTT were designated as tissue *KRAS*‐positive.

### Statistical analysis

The chi‐square test was used for statistical analyses. These were conducted using JMP version 14.2 software (SAS Institute Inc.).

## RESULTS

NGS analysis was performed on blood samples in 214 patients (Figure S1). Tissue samples analyzed with Oncomine DxTT could be retrieved for 147 of these patients. *KRAS* mutation was detected in 28 (19.0%) patients. *KRAS* mutations were detected in blood samples from 33 patients (15.4%), of whom 31 (14.5%) had a histopathological diagnosis. The characteristics of liquid *KRAS*‐positive 33 patients are shown in Table 1. The median age of liquid *KRAS*‐positive patients was 68.5 (95% CI: 24–82) years, 23 (69.7%) were male, and 25 (75.8%) were smokers. The histological type was adenocarcinoma in 27 cases (81.8%). Of the 27 liquid *KRAS‐*positive patients with adenocarcinoma, 25 were diagnosed histologically, seven (28.0%) had mucinous adenocarcinoma and 18 (72.0%) had nonmucinous adenocarcinoma. The *KRAS* mutation positivity rate by tissue was 35.7% in patients with mucinous adenocarcinoma and 50.0% in patients with nonmucinous adenocarcinoma (data not shown).

**TABLE 1:** Characteristics of the study patients.

	NGS testing of liquid samples		Liquid *KRAS*‐positive	
	*N* = 214		*N* = 33	
Age (range)	70.5 (24–86)		68.5 (24–82)	
Sex, *N* (%)				
Male	140	(65.4)	23	(69.7)
Female	74	(34.6)	10	(30.3)
Smoking status, *N* (%)				
Current or former	158	(73.8)	25	(75.8)
Never	56	(26.2)	8	(24.2)
Histology, *N* (%)				
Adeno	159	(74.2)	27	(81.8)
Mucinous Ad	15	(7.0)	7	(21.2)
Nonmucinous Ad	144	(67.3)	20	(60.6)
Sq	42	(19.6)	4	(12.1)
NOS	10	(4.7)	2	(6.1)
Other	3	(1.4)	0	
Stage (UICC eighth edition), *N* (%)				
I–II	4	(1.9)	1	(3.0)
III	15	(7.0)	8	(24.2)
IV or recurrence	195	(91.1)	24	(72.7)
Sites of metastases				
Lung	42	(19.6)	5	(15.1)
Pleura	67	(31.3)	11	(33.3)
Adrenal gland	23	(10.7)	7	(21.2)
Muscle	13	(6.0)	7	(21.2)
Brain	36	(16.8)	6	(18.2)
Bone	76	(35.5)	17	(51.5)
Skin	8	(3.7)	4	(12.1)
Liver	22	(10.3)	3	(9.1)

Abbreviations: Ad, adenocarcinoma; NOS, not otherwise specified; Sq, squamous cell carcinoma; UICC, Union for International Cancer Control.

There were no major differences between the liquid *KRAS*‐positive and *KRAS‐*negative groups in age, gender, smoking history, or stage (Table 1). Overall, bone metastasis was the most common metastatic lesion, followed by pleural dissemination. Skin and muscle metastasis tended to be more common in *KRAS*‐positive cases.

Among adenocarcinomas, the proportion of mucinous adenocarcinomas tended to be more frequent among *KRAS*‐positive patients. There was a difference in detection rate between tissue and blood samples for tissue type (Tables 2 and 3). Among 31 patients who had a histopathological confirmed diagnosis, there were 10 patients with positive blood samples only and 21 with positive blood and tissue samples. Adenocarcinoma was the most common histological type in both groups. Mucinous adenocarcinoma accounted for 28.6% of patients with positive blood and tissue samples, 10.0% of patients with positive blood samples only, and 57.1% of patients with positive tissue samples only. Mucinous adenocarcinoma tended to be less common in the group with positive blood samples. There were no obvious differences in clinical background between the two groups.

**TABLE 2:** Characteristics of *KRAS* mutations by biopsy type with histopathology.

	*KRAS* mutation detected in liquid and tissue samples		*KRAS* mutation detected only in liquid samples		*KRAS* mutation detected only in tissue samples	
	(*N* = 21)		(*N* = 10)		(*N* = 7)	
Age (range)	67.5 (34–80)		70.0 (24–82)		75.0 (60–86)	
Sex, *N* (%)						
Male	12	(57.1)	9	(90)	6	(85.7)
Female	9	(42.9)	1	(10)	1	(14.3)
Smoking status, *N* (%)						
Current or former	15	(71.4)	8	(80)	5	(71.4)
Never	6	(28.6)	2	(20)	2	(28.6)
Histology, *N* (%)						
Non‐Ad	4	(19.1)	2	(20)	0	
Ad	17	(81.0)	8	(80)	7	(100)
Mucinous Ad	6	(28.6)	1	(10)	4	(57.1)
Nonmucinous Ad	11	(52.4)	7	(70)	3	(42.9)
Stage (UICC eighth edition), *N* (%)						
I–II	0		1	(10)	1	(14.3)
III	6	(28.6)	2	(20)	2	(28.6)
IV or recurrence	15	(71.4)	7	(70)	4	(57.1)
Sites of metastases						
Lung	3	(14.3)	2	(20)	3	(42.9)
Other						
Bone	9	(42.9)	8	(80)	0	
Pleura	7	(33.3)	2	(20)	0	
Adrenal gland	5	(23.8)	2	(20)	0	
Muscle	4	(19.1)	2	(20)	0	
Brain	4	(19.1)	2	(20)	1	(14.3)
Skin	1	(4.8)	1	(10)	0	
Liver	1	(4.8)	1	(10)	0	

Abbreviation: Ad, adenocarcinoma; UICC, Union for International Cancer Control.

**TABLE 3:** *KRAS* mutation status.

	*KRAS* mutation detected in liquid and tissue samples		*KRAS* mutation detected only in liquid samples		*KRAS* mutation detected only in tissue samples	
	(*N* = 21)		(*N* = 10)		(*N* = 7)	
*KRAS* mutation, N (%)						
G12V	7	(33.3)	1	(10)	3	(42.9)
G12A	4	(19.0)	1	(10)	1	(14.3)
G12D	3	(14.3)	2	(20)	2	(28.6)
G12C	3	(14.3)	1	(10)	1	(14.3)
Q61H	3	(14.3)	1	(10)	0	
G12S	0		2	(20)	0	
G13D	1	(4.8)	0		0	
A146T	0		1	(10)	0	
Amplification	0		1	(10)	0	

Among 31 liquid *KRAS*‐positive patients, G12V was the most common mutation (*n* = 8, 25.8%). G12C was observed in four patients (12.9%). There were no differences between liquid *KRAS*‐positive and tissue *KRAS*‐positive patients in *KRAS* mutation type or frequency.

Metastatic sites and the *KRAS* mutation positive rate in nonmucinous and mucinous adenocarcinoma are shown in Table 4. Nonmucinous and mucinous adenocarcinoma also tended to have higher rates of distant metastasis among patients with positive blood samples only than in patients with positive tissue samples only. A higher percentage of mucinous adenocarcinoma were associated with muscle metastasis compared with nonmucinous adenocarcinoma. Only one patient (5.3%) with *KRAS*‐positive nonmucinous adenocarcinoma and lung metastasis had a positive blood sample. Among patients with mucinous adenocarcinoma, only three patients (30%) had positive tissue samples. In patients with lung metastasis only, blood and tissue samples had different rates of *KRAS* mutations.

**TABLE 4:** Sites of metastasis and *KRAS* mutation positivity rate in nonmucinous and mucinous adenocarcinoma.

Sites of metastasis, *N* (%)	Nonmucinous adenocarcinoma				Mucinous adenocarcinoma			
	Liquid *KRAS*‐positive		Tissue *KRAS*‐positive		Liquid *KRAS*‐positive		Tissue *KRAS*‐positive	
	*N* = 18		*N* = 14		*N* = 7		*N* = 10	
Lung	1	(5.6)	1	(7.1)	1	(14.3)	3	(30)
Lung only	1	(5.6)	0		0		3	(30)
Other								
Bone	9	(50)	3	(21.4)	5	(71.4)	4	(40)
Pleura	6	(33.3)	3	(21.4)	3	(42.9)	3	(30)
Adrenal gland	4	(22.2)	2	(14.3)	2	(28.6)	2	(20)
Muscle	1	(5.6)	0		5	(71.4)	4	(40)
Brain	3	(16.7)	3	(21.4)	3	(42.9)	2	(20)
Skin	1	(5.6)	1	(7.1)	1	(14.3)	0	
Liver	3	(16.7)	1	(7.1)	0		1	(10)

## DISCUSSION

In this study, we examined the characteristics of liquid *KRAS*‐positive NSCLC. We reported liquid *KRAS*‐positive NSCLC was different from *KRAS*‐positive tested by NGS with tissue samples (tissue *KRAS*‐positive) between mucinous adenocarcinoma and nonmucinous adenocarcinoma. In this study, *KRAS* gene mutation frequency in blood samples was 14.5% in NSCLC cases and 17.0% in lung adenocarcinoma cases. Regarding *KRAS* mutation subtypes, the frequency of the G12C mutation was 12.9%. *KRAS*‐positive patients were more likely to have mucinous adenocarcinoma compared to *KRAS‐*negative patients in the blood sample–positive and tissue sample–positive groups, respectively.

The *KRAS* mutation detection rate in blood and tissue samples might differ among patients with mucinous and nonmucinous adenocarcinoma. The percentage of *KRAS* positive cases by histology based on liquid samples was 22.6% (7 of 31) in patients with mucinous adenocarcinoma and 58.1% (18 of 31) in patients with nonmucinous adenocarcinoma. In addition, the *KRAS* mutation detection frequencies were 35.7% in patients with mucinous adenocarcinoma and 50.0% in patients with nonmucinous adenocarcinoma (data not shown). Based on liquid samples, the prevalence of mucinous adenocarcinoma tended to be lower. Badi et al. reported that in a review of 1655 cases of lung adenocarcinoma, *KRAS* mutations were reported to be present in 27% of cases.^17^ In the MSK‐Impact study, the *KRAS* mutation positivity rate was 33.2% in 1586 lung adenocarcinoma cases.^18^ From Japan, the prevalence of *KRAS* mutations based on Oncomine DxTT with tissue specimens was reported to be 14.1% in lung adenocarcinoma cases.^19^ The *KRAS* positivity rate was reported to be 14% in LC‐SCRUM‐Asia.^20^ Previous studies have found that levels of ctDNA are frequently low or less sensitive in CNS tumors and mucinous tumors.^13^, ^14^ Nonmucinous adenocarcinoma is more likely to be detected with liquid NGS than mucinous adenocarcinoma.^14^ The lower *KRAS* mutation positivity rate with liquid samples might be the reason for the low detection rate.

Hematogenous spread is the most common mechanism for intrapulmonary metastasis. Hematogenous lung metastasis is the result of the migration of malignant cells from the pulmonary venous circulation into the systemic circulation. Hematogenous spread to the lung is associated with an increased risk of distant metastasis.^21^ In lung adenocarcinoma, discontinuous, noninvasive neoplastic foci, which are the histological surrogate of aerogenous spread, are more commonly seen in mucinous neoplasms.^22^ There is a difference in the form of metastasis between mucinous and nonmucinous adenocarcinoma. Hematogenous metastasis is associated with nonmucinous adenocarcinoma while transairway metastasis is associated with mucinous adenocarcinoma. This difference might have affected mutation positivity rates. In the LC‐SCRUM‐Liquid study,^23^ which evaluated plasma cell‐free DNA and tissue‐based sequencing concordance for comprehensive oncogenic driver detection in NSCLC, the *KRAS* mutation detection rate was 12.1%. In that study, the analysis of the effect on tissue type did not include mucinous adenocarcinoma or metastatic disease. Although the study involved colorectal cancer, the ctDNA detection rate of ctDNA was reported to be low in patients with lung metastasis only in SCRUM‐Japan GOZILA.^24^ A previous study showed a relationship between *KRAS* mutations and mucinous differentiation in cancers of various human organs, including colorectal and lung cancer.^25^, ^26^ The *KRAS* mutation detection rate in liquid samples might be different for metastatic disease. In this study, there were four *KRAS*‐positive patients with only lung metastasis. Of these patients, only one was diagnosed with nonmucinous adenocarcinoma and only one patient was liquid *KRAS*‐positive. The three other patients with lung metastases only were diagnosed with mucinous adenocarcinoma and *KRAS* mutations were detected only in tissue samples among these patients. They all had airway metastases, not hematogenous metastases. Therefore, *KRAS* mutations might not have been detected in blood specimens. In patients with mucinous adenocarcinoma and only pulmonary metastases, analysis of blood specimens is likely to be false‐negative and should be carefully handled by attempting analysis on tissue samples. Regarding the frequency of *KRAS* mutations in Western countries, the G12C mutation was the most common (40%–50%), followed by G12V (20%–25%).^23^ In LC‐SCRUM‐Asia, the most common *KRAS* mutation was G12C (29.2%), followed by G12D (23.4%) and G12V (20.7%).^24^ The types of *KRAS* mutations observed in blood samples from this study were the same as in previous studies.^23^, ^24^ Of note, 12.9% of mutations were G12C. This is low compared to data from overseas, but consistent with reports from Asia but less so from Japan.^24^, ^25^, ^26^ Consideration of tissue type may increase the detection rate of *KRAS* mutations.

The present study had limitations, such as the inability to eliminate bias, especially in patient selection. The possibility of selection bias and the incompleteness of NGS analysis of tissue samples in all participants might have influenced the interpretation of the study results. It was also a single center study with a small number of participants. NGS analysis was not available for tissue samples in all patients. Genetic abnormalities detected in blood samples might represent clonal hematopoiesis.

In conclusion, the targets for *KRAS*‐positive lung cancer that can be detected in blood and tissue samples differ. In patients with mucinous adenocarcinoma and lung metastasis only, the detection rate might be poor for blood specimens. To improve practice, it is important to consider these differences when evaluating indications for testing.

## AUTHOR CONTRIBUTIONS

All authors had full access to the data in the study and take responsibility for integrity of the data and the accuracy of the data analysis. Conceptualization, Y.A, A.K and M.N; Investigation, Y.A, A.K, S.K.L and H.N; Formal analysis, Y.A and A.K; Resources, Y.A, A.K and M.N; Writing‐original draft, Y.A and M.N; Writing‐review and editing, Y.A, S.K.L, H.N, A.K, K.M, S.K, T.A, R.T, R.A, K.U, S.K, N.Y and M.N; Visualization, Y.A; Supervision, M.N.

## CONFLICT OF INTEREST STATEMENT

Dr Nishio received honoraria from Novartis, ONO Pharmaceutical, Chugai Pharmaceutical, Bristol‐Myers Squibb, TAIHO Pharmaceutical, Eli Lilly, Pfizer, Astellas Pharma, Daiichi Sankyo, MSD, Abbvie, Takeda Pharmaceutical, Boehringer Ingelheim, Nippon Kayaku, Merck, Janssen and AstraZeneca. Dr Yanagitani received honoraria from Chugai Pharmaceutical, ONO Pharmaceutical, Bristol‐Myers Squibb, AstraZeneca, Eli Lilly, Pfizer and Takeda Pharmaceutical. Dr Kitazono received honoraria from AstraZeneca, Chugai Pharmaceutical, ONO Pharmaceutical, Pfizer. Dr. Siew‐Kee Low received consulting fees from Cancer Precision Medicine Inc. and honoraria from illumina and Thermo Fisher. Dr Hironori Ninomiya received honoraria from Daiichi Sankyo, Thermo Fisher and Eli Lilly. Dr Uchibori received honoraria from Astra Zeneca, Amgen, Chugai Pharmaceutical, Takeda Pharmaceutical, ONO Pharmaceutical, Novartis, Eli‐Lilly, Thermo Fisher, Bristol Myers Squib, Merck and Daiichi‐Sankyo. Dr Ariyasu received honoraria from Astra Zeneca, Chugai and Bristol Myers Squib. All the other authors have stated that they have no conflicts of interest.

## Supporting information


**FIGURE S1.** Study flow.

## References

[1] Islami F , Ward EM , Sung H , Cronin KA , Tangka FKL , Sherman RL , et al. Annual report to the nation on the status of cancer, part 1: National Cancer Statistics. J Natl Cancer Inst. 2021;113(12):1648–1669. 34240195 10.1093/jnci/djab131 PMC8634503

[2] Howlader N , Forjaz G , Mooradian MJ , Meza R , Kong CY , Cronin KA , et al. The effect of advances in lung‐cancer treatment on population mortality. N Engl J Med. 2020;383(7):640–649. 32786189 10.1056/NEJMoa1916623 PMC8577315

[3] Stehelin D , Varmus HE , Bishop JM , Vogt PK . DNA related to the transforming gene(s) of avian sarcoma viruses is present in normal avian DNA. Nature. 1976;260(5547):170–173. 176594 10.1038/260170a0

[4] Luo J , Ostrem J , Pellini B , Imbody D , Stern Y , Solanki HS , et al. Overcoming KRAS‐mutant lung cancer. Am Soc Clin Oncol Educ Book. 2022;42:1–11. 10.1200/EDBK_360354 35412860

[5] Riely GJ , Marks J , Pao W . KRAS mutations in non‐small cell lung cancer. Proc Am Thorac Soc. 2009;6(2):201–205. 19349489 10.1513/pats.200809-107LC

[6] Skoulidis F , Li BT , Dy GK , Price TJ , Falchook GS , Wolf J , et al. Sotorasib for lung cancers with KRAS p.G12C mutation. N Engl J Med. 2021;384(25):2371–2381. 34096690 10.1056/NEJMoa2103695 PMC9116274

[7] Jänne PA , Riely GJ , Gadgeel SM , Heist RS , Ou SHI , Pacheco JM , et al. Adagrasib in non–small‐cell lung cancer harboring a KRASG12C mutation. N Engl J Med. 2022;387(2):120–131. 35658005 10.1056/NEJMoa2204619

[8] Yu HA , Sima CS , Shen R , Kass S , Gainor J , Shaw A , et al. Prognostic impact of KRAS mutation subtypes in 677 patients with metastatic lung adenocarcinomas. J Thorac Oncol. 2015;10(3):431–437. 25415430 10.1097/JTO.0000000000000432 PMC4479120

[9] Nicholson AG , Tsao MS , Beasley MB , Borczuk AC , Brambilla E , Cooper WA , et al. The 2021 WHO classification of lung tumors: impact of advances since 2015. J Thorac Oncol. 2022;17(3):362–387. 34808341 10.1016/j.jtho.2021.11.003

[10] Travis WD , Brambilla E , Noguchi M , Nicholson AG , Geisinger KR , Yatabe Y , et al. International association for the study of lung cancer/american thoracic society/european respiratory society international multidisciplinary classification of lung adenocarcinoma. J Thorac Oncol. 2011;6(2):244–285. 21252716 10.1097/JTO.0b013e318206a221 PMC4513953

[11] Liam CK , Mallawathantri S , Fong KM . Is tissue still the issue in detecting molecular alterations in lung cancer? Respirology. 2020;25(9):933–943. 32335992 10.1111/resp.13823

[12] Rolfo C , Mack P , Scagliotti GV , Aggarwal C , Arcila ME , Barlesi F , et al. Liquid biopsy for advanced NSCLC: a consensus statement from the International Association for the Study of Lung Cancer. J Thorac Oncol. 2021;16(10):1647–1662. 34246791 10.1016/j.jtho.2021.06.017

[13] Siravegna G , Mussolin B , Venesio T , Marsoni S , Seoane J , Dive C , et al. How liquid biopsies can change clinical practice in oncology. Ann Oncol. 2019;30(10):1580–1590. 31373349 10.1093/annonc/mdz227

[14] Bettegowda C et al. Detection of circulating tumor DNA in early‐ and late‐stage human malignancies. Sci Transl Med. 2014;6(224):224ra24. 10.1126/scitranslmed.3007094 PMC4017867 24553385

[15] Low S‐K , Ariyasu R , Uchibori K , Hayashi R , Chan HT , Chin YM , et al. Rapid genomic profiling of circulating tumor DNA in non‐small cell lung cancer using Oncomine precision assay with Genexus TM integrated sequencer. Transl Lung Cancer Res. 2022;11:711–721. 35693289 10.21037/tlcr-21-981 PMC9186171

[16] Administration., F.a.D . Oncomine™Dx Target Test Part I:Sample Preparation and Quantification User Guide. Revision C02017. 2017.

[17] El Osta B , Behera M , Kim S , Berry LD , et al. Characteristics and outcomes of patients with metastatic KRAS‐mutant lung adenocarcinomas: the lung cancer mutation consortium experience. J Thorac Oncol. 2019;14(5):876–889. 30735816 10.1016/j.jtho.2019.01.020 PMC8108452

[18] Schoenfeld AJ , Rizvi H , Bandlamudi C , Sauter JL , Travis WD , Rekhtman N , et al. Clinical and molecular correlates of PD‐L1 expression in patients with lung adenocarcinomas. Ann Oncol. 2020;31(5):599–608. 32178965 10.1016/j.annonc.2020.01.065 PMC7523592

[19] Sakata S , Otsubo K , Yoshida H , Ito K , Nakamura A , Teraoka S , et al. Real‐world data on NGS using the Oncomine DxTT for detecting genetic alterations in non‐small‐cell lung cancer: WJOG13019L. Cancer Sci. 2022;113(1):221–228. 34704312 10.1111/cas.15176 PMC8748216

[20] Matsumoto S , Ikeda T , Yoh K , Sugimoto A , Kato T , Kunimasa K , et al. Impact of rapid multigene assays with short turnaround time (TAT) on the development of precision medicine for non‐small cell lung cancer (NSCLC). J Clin Oncol. 2021;39(15_suppl):9094.

[21] Chambers AF , Groom AC , Macdonald IC . Dissemination and growth of cancer cells in metastatic sites. Nat Rev Cancer. 2002;2(8):563–572. 12154349 10.1038/nrc865

[22] Gaikwad A , Souza CA , Inacio JR , Gupta A , Sekhon HS , Seely JM , et al. Aerogenous metastases: a potential game changer in the diagnosis and management of primary lung adenocarcinoma. AJR Am J Roentgenol. 2014;203(6):W570–W582. 25415722 10.2214/AJR.13.12088

[23] Burns TF , Borghaei H , Ramalingam SS , Mok TS , Peters S . Targeting *KRAS*‐mutant non–small‐cell lung cancer: one mutation at a time, with a focus on *KRAS* G12Cmutations. J Clin Oncol. 2020;38(35):4208–4218. 33104438 10.1200/JCO.20.00744 PMC7723684

[24] Tamiya Y , Zenke Y , Matsumoto S , Furuya N , Sakamoto T , Kato T , et al. Therapeutic impact of mutation subtypes and concomitant STK11 mutations in KRAS–mutated non‐small cell lung cancer (NSCLC): a result of nationwide genomic screening project (LC‐SCRUM‐Japan). J Clin Oncol. 2020;38(15 suppl):9589.

[25] Jia Y , Jiang T , Li X , Zhao C , Zhang L , Zhao S , et al. Characterization of distinct types of KRAS mutation and its impact on first‐line platinum‐based chemotherapy in Chinese patients with advanced non‐small cell lung cancer. Oncol Lett. 2017;14:6525–6532. 29163686 10.3892/ol.2017.7016 PMC5686437

[26] Zheng D , Chen H , Wang R , Zhang Y , Pan Y , Cheng X , et al. The prevalence and prognostic significance of KRAS mutation subtypes in lung adenocarcinomas from Chinese populations. Onco Targets Ther. 2016;9:833–843. 26955281 10.2147/OTT.S96834 PMC4768896

