# Supplementary material for: Clinical characteristics of patients with KRAS mutation detected by liquid biopsy
Source: Thorac Cancer. 2023 Sep 26;14(33):3317–22. doi: 10.1111/1759-7714.15123 (PMC10665783; doi:10.1111/1759-7714.15123)
Supplement: Supplementary file 1 — FIGURE S1. Study flow. [file TCA-14-3317-s001.docx]

Supplementary Figure 1. Study flow

NGS of liquid samples

N=214

Liquid *KRAS* mutation–positive NSCLC

N=33

Tissue-based pathological diagnosis

N=31

NGS, next-generation sequencing; NSCLC, non–small cell lung cancer.
